# SOCIAL ENGAGEMENT IS ASSOCIATED WITH COGNITION IN OLDER ADULTS FROM UNDERSERVED QUILOMBOLA COMMUNITIES IN BRAZIL

**DOI:** 10.1093/geroni/igae098.2880

**Published:** 2024-12-31

**Authors:** Sharon Sanz Simon, Carolina Cappi, João Cabral Junior, Gilberto Alves, Bruno Carneiro Alves de Oliveira

**Affiliations:** Columbia University, New Rochelle, New York, United States; Icahn School of Medicine at Mount Sinai, New York, New York, United States; Federal University of Maranhão, Pinheiro, Maranhao, Brazil; Faculty of Medicine, Federal University of Maranhã, São Luís, Maranhao, Brazil; Federal University of Maranhão, São Luis, Maranhao, Brazil

## Abstract

Quilombos are settlements founded by descendants of runaway slaves in Brazil, typically located in rural areas. Quilombola communities often present poor basic sanitation, high levels of illiteracy, and limited access to social and health services. Although the Quilombola population is socially vulnerable and likely to present an increased risk for dementia, it is underrepresented in aging research. The present study aims to investigate the association between cognition and socio-cultural engagement, potentially protective for dementia. Method: This cross-sectional study was conducted in 11 Quilombola communities in Maranhão, Brazil. The study comprised 221 older adults (60-104 years), selected in partnership with local services and community health workers. Participants completed a health survey and a cognitive screening. Demographics, cardiovascular risk factors (CRF), cognitive performance, and social engagement were described. Regression analysis examined the association between social engagement and cognition, controlling for age, sex, race, education, family income, and cardiovascular risk factors. We also examined moderation effects of sex and age on the regressions.

**Results:**

Higher social engagement was associated with better cognition (p <.001) above and beyond demographics and CRF. We observed the social activities that mostly drove the results were attending “community meetings” and “religious/faith services”. Age and sex did not moderate the associations.

**Conclusion:**

Social engagement may be a key aspect of cognitive/brain health in Quilombola population. Social engagement may not only provide social/emotional support, but also facilitate access to basic needs. Although the results are limited by the cross-sectional design and survival bias, it may inform strategies in underserved populations.

